# Draft Genome Sequence of a Novel Acidophilic Iron-Oxidizing *Firmicutes* Species, “*Acidibacillus ferrooxidans*” (SLC66^T^)

**DOI:** 10.1128/genomeA.00383-16

**Published:** 2016-05-19

**Authors:** Ivan Ñancucheo, Renato Oliveira, Hivana Dall’Agnol, D. Barrie Johnson, Barry Grail, Roseanne Holanda, Gisele Lopes Nunes, Sara Cuadros-Orellana, Guilherme Oliveira

**Affiliations:** a Vale Institute of Technology, Belém, Pará, Brazil; b Facultad de Ingeniería y Tecnología, Universidad San Sebastián, Concepción, Chile; c Universidade Federal do Maranhão, São Luís, Maranhão, Brazil; d College of Natural Sciences, Bangor University, Bangor, United Kingdom; e René Rachou Research Center, Fiocruz, Belo Horizonte, Minas Gerais, Brazil

## Abstract

Here, we present the draft genome sequence of the type strain of “*Acidibacillus ferrooxidans*,” a mesophilic, heterotrophic, and acidophilic bacterium that was isolated from mine spoilage subjected to accelerated weathering in humidity cell tests carried out by the former U.S. Bureau of Mines in Salt Lake City, UT.

## GENOME ANNOUNCEMENT

Microorganisms that have an optimum pH for growth of <3 are classed as extreme acidophiles; many of these contribute to the biogeochemical cycling of iron and sulfur in these environments. Acidophilic bacteria, as a group, vary significantly in how they assimilate carbon. Many species are obligate autotrophic, others are obligate heterotrophic, and some (facultative autotrophs) fix inorganic carbon (CO_2_) even though they preferentially assimilate organic carbon (1). Some species of acidophilic prokaryotes, mostly notably iron-oxidizing *Acidithiobacillus* and *Leptospirillum* spp. (*Proteobacteria*), are responsible for the bioleaching of sulfide minerals. Known species of Gram-positive acidophilic bacteria are affiliated with two phyla, *Firmicutes* and *Actinobacteria*, and include species that are facultative autotrophs and obligate heterotrophs. Here, we report the draft genome sequence of strain SLC66^T^, the type strain of the novel species “*Acidibacillus ferrooxidans*” (2, 3). This acidophile had been isolated from mine waste regolithic material subjected to accelerated weathering in humidity cells at pH 2.9 and 25°C (4, 5). *A. ferrooxidans* is one of two proposed species of the novel genus “*Acidibacillus*,” the other being “*A. sulfuroxidans*.” *Acidibacillus* is the third genus of acidophilic *Firmicutes* to be described, the others being *Alicyclobacillus* and *Sulfobacillus*. *A. ferrooxidans* is classified as facultative chemolithoheterotroph and can obtain energy from both inorganic and organic electron donors, but it requires an organic carbon source, with complex organic materials (e.g., yeast extract) being the most effective carbon. *A. ferrooxidans* is mesophilic and acidophilic (growth optimal at 30°C and pH 2.9), in contrast to the moderate thermophile *A. sulfuroxidans* (growth optimal at 43°C and pH 1.8). *A. ferrooxidans* catalyzes the dissimilatory oxidation of ferrous iron (and therefore the oxidative dissolution of sulfidic minerals, such as pyrite), while *A. sulfuroxidans* oxidizes both reduced iron and sulfur. Both species can couple the oxidation of organic carbon with the reduction of ferric iron in the absence of oxygen.

Sequencing of the genome of SLC66^T^ resulted in 2,122,638 paired-end reads obtained using the Illumina MiSeq platform, with 34.7× coverage of the expected genome size. The reads were trimmed and filtered using the Fastx Toolkit (http://hannonlab.cshl.edu/fastx_toolkit/), with a Phred score of 20, resulting in a total of 1,996,872 reads. The assembly of the genome sequence was performed with the Roche Newbler assembler version 2.9, ABySS 1.9 (6), and IDBAUD 1.1.1 (7). The contigs were integrated using the MIX (8) pipeline, resulting in a total of 175 contigs. The draft genome of strain SCL66^T^ comprises a single chromosome of 3.2 Mb, with an overall G+C content of 51.97%. PATRIC (9) was used for automated annotation and identification of rRNA and tRNA, along with RNAmmer (10) and tRNAscan-SE (11), respectively. The draft genome harbored 4 rRNA genes, 53 tRNA genes, and a total of 3,497 protein-coding genes, from which 1,830 are genes with known functions and 1,667 are genes coding for hypothetical proteins.

### Nucleotide sequence accession numbers.

This whole-genome shotgun project has been deposited at GenBank under the accession no. LVKL00000000. The version described in this paper is version LVKL00000000.1.
